# Long-term variability of air quality and greenhouse gas emissions from rice crop burning in Punjab during 2012–2020

**DOI:** 10.1039/d5ra09439a

**Published:** 2026-03-10

**Authors:** Harsimranjit Kaur Romana, Dericks Praise Shukla, Ramesh P. Singh

**Affiliations:** a DExtER Lab, School of Civil and Environmental Engineering, Indian Institute of Technology (IIT) Mandi Himachal Pradesh 175005 India dericks@iitmandi.ac.in; b School of Life and Environmental Sciences, Schmid College of Science and Technology, Chapman University Orange California 92866 USA

## Abstract

Punjab, India's primary rice and wheat production hub, has witnessed rapid expansion of paddy cultivation over the past two decades, driven by minimum support price incentives, changes in government policies, alignment of sowing with the monsoon season and the adoption of high-yielding varieties. This transition has intensified groundwater extraction and shortened the fallow period between rabi and kharif crop seasons, reducing the window between rice harvesting and wheat sowing, leading to widespread open-field burning of rice residue and recurrent post-monsoon air-quality deterioration across the Indo-Gangetic Plain. Despite numerous short-term or single-pollutant assessments, a spatially resolved, multi-pollutant and multi-decadal evaluation linking crop production, fire activity, satellite observations, and future emission trajectories remains limited. In this work, we presented a comprehensive district- and grid-resolved emission inventory for crop residue burning in Punjab for 2000–2020 and integrated it with satellite-derived atmospheric indicators, active fire counts, and scenario-based forecasting. The study quantified particulate (PM_2.5_, PM_10_, black carbon, and organic carbon), gaseous (SO_2_, CO, NO_*x*_, and NH_3_), toxic organic (NMVOCs and PAHs), and greenhouse gas (CO_2_, CH_4_, and N_2_O) emissions and evaluated their consistency with satellite observations of aerosol optical depth and trace gases. Rice cultivation expanded from ∼0.26 to ∼3.14 million hectares during 2000–2020, accompanied by substantial yield gains, which translated into marked growth in residue generation and emissions. The Malwa region consistently emerged as the dominant multi-pollutant hotspot, whereas Doaba and Majha exhibited lower but rising burdens. Strong statistical relationships between fire counts, estimated emissions, and satellite observations (*R*^2^ ≈ 0.60–0.81) confirm the robustness of the inventory and demonstrate a direct linkage between residue burning intensity and atmospheric loading. We tried to couple the long-term agricultural production trends with multi-pollutant emission quantification, satellite validation, and grid-wise spatial analysis and explored the scenario-based future projections based on the percentage of residue burnt. Scenario-based forecasts for 2040 indicated that continuation of high residue-burning fractions could lead to substantial increases in pollutant concentrations, particularly in central and south-western districts such as Ferozpur, Hoshiarpur, and Rupnagar. The results establish crop residue burning as a persistent, spatially concentrated emission source, emphasizing the need for location-specific residue management strategies rather than state-specific steps to achieve simultaneous air-quality improvement.

## Abstract 2

Punjab, India's main rice-producing state, has seen rapid expansion of paddy cultivation over past two decades, driven by monsoon-aligned sowing, adoption of high-yielding varieties, minimum support price incentives and shifts in government policy.

## Graphical abstract

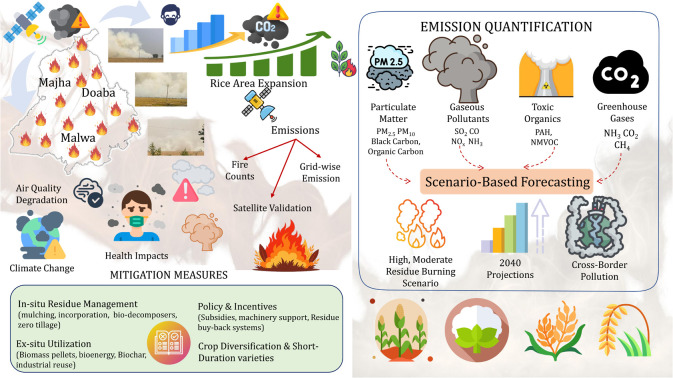

## Introduction

The Green Revolution emerged to curb the alarming scarcity of food worldwide following World War II. The high-yielding varieties of rice, wheat and maize were mainly introduced in developing countries (India, Mexico, Bangladesh, Indonesia and China).^1^ These new high-yielding crops helped to improve yields to avoid famine conditions during the post-independence period in the Indian subcontinent.^2,3^ As a result, India experienced a transcendent shift in agricultural production. The output of wheat increased from 8.36 million tonnes in 1950 to 44.76 million tonnes in 1989 (435.40% increase), and the output of rice increased from 26.29 million tonnes in 1950 to 59.78 million tonnes in 1989 (127.38% increase).^4^ This helped the economic growth of the country alongside food security. However, these semi-dwarf, high-yielding varieties require high amounts of fertilizers, pesticides/insecticides and water. Consequently, the intensive and unsustainable use of these inputs caused overexploitation of groundwater, soil degradation and poor surface and groundwater quality.^5,6^ Therefore, the agricultural boom came with an ecological and environmental bane.

Furthermore, farmers started using mechanized harvesting in 1986 following the Red Revolution, instead of manual harvesting, leaving about few feet residue in the field.^7,8^ This posed a new problem of crop residue management. The lack of labour and small time-gap between two crop seasons led farmers to find an economic way to clear and prepare the fields for the next crop: open burning of the crop residue.^8–10^

The crop residue burning in the high rice yielding states such as Punjab, Haryana, and Uttar Pradesh causes severe air pollution and smog across many cities of IGP (Indo-Gangetic Plain). It is estimated that nearly 87 Mt residue was burnt in the country, out of which, 9 Mt was burnt in Punjab alone.^11–13^ In addition to the air pollution caused, stubble burning causes deterioration of soil quality. For instance, organic carbon is oxidised to form carbon dioxide, nitrogen content is converted to nitrate and microbial community is destroyed in the topsoil (2.5 cm).^14,15^ It is estimated by Punjab Agriculture University, that, nearly 0.824 million tonnes of NPK (Nitrogen Phosphate Potassium) are lost annually from the fertile soil of the state.

Open stubble burning has currently emerged as the primary source of aerosol particles and second largest sources of trace gases.^16,17^ The massive amounts of emissions loaded into the atmosphere lead to radiation imbalance, albedo, and cloud condensation nuclei concentrations, and can change the environmental chemistry, affecting the air quality of the area. Moreover, carbon aerosols emitted can scatter solar radiation, thus contributing to atmospheric heating. Subsequently, this affects cryosphere snow deposits and glaciers. In addition, the gaseous pollutants contribute to secondary aerosols and ground-level ozone. This further degrades the air quality. The stubble burning emits various trace gases such as particulate matter (PM_2.5_ and PM_10_), carbon dioxide (CO_2_), carbon monoxide (CO), sulphur dioxide (SO_2_), nitrogen oxides (NO_*x*_), ammonia (NH_3_), methane (CH_4_), volatile organic compounds (VOCs), elemental carbon (EC), organic carbon (OC), black carbon (BC), polycyclic aromatic hydrocarbons (PAHs) and non-methane volatile organic compounds (NMVOCs).^18^ Particulate matter and carbonaceous aerosols can penetrate deep into the lungs and have an adverse impact on human health.^19^ Exposure to these emissions can cause various chronic health ailments such as asthma, heart disease, stroke and early mortality. According to the World Health Organization (WHO), seven million deaths are caused by poor air quality worldwide in 2012.^20^

Moreover, the emitted trace gases and aerosol particles have a long-range transport, as they remain suspended in the atmosphere for days to weeks. Thus, regional open fire can lead to air pollution in the central and south-eastern parts of India.^21^ During the rice crop residue-burning period in September to November in Punjab, the Delhi NCR region becomes a “smog chamber” due to the unfavourable meteorological parameters such as low wind speed and high relative humidity. The impacts of burning are also observed up to the eastern parts of the Indo-Gangetic Plain (IGP).^22^ In other words, the air quality index in IGP becomes very poor and severe at few places. The particulate matter concentration during the rabi harvest season is approximately 5–10 times more than the WHO standard, which escalates air pollution and health effects due to the intensification.

Literature suggests that there are numerous mitigation measures based on adsorbents such as activated carbon-based adsorbents, biochars, wet scrubbers, and photocatalytic adsorbents. However, *in situ* or *ex situ* residue management is a reliable option to control residue burning and subsequently the pollution.^23–25^

In recent years, efforts have been made to publish many aspects (emissions, air quality, meteorological and atmospheric parameters, mixing of aerosols, impacts on visibility, fog formation, and health impacts) of crop residue burning. However, a long-term analysis of emissions has not been attempted. The present study focuses on the increase in rice production during the past 20 years (2000–2020), and consequently, on the rise in air pollution using the emission factor of rice crop residue burning. The objectives of the study are as follows:

* Assess impacts of crop residue burning on air quality for the past 20 years.

* To correlate particulate matter and active fire counts in the region.

* Establish correlation between satellite-observed and estimated air pollutants.

* Forecast pollutant emissions for three different scenarios (80% residue burnt, 50% residue burnt and 30% residue burnt).

## Study area, data used and methodology

**Fig. 1:**
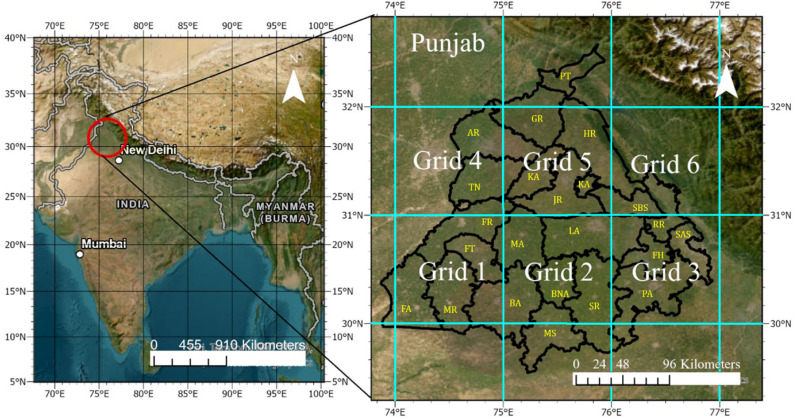
Location of the study area showing various districts of Punjab and the grid division for the study area. Different districts are PT (Pathankot), GR (Gurdaspur), AR (Amritsar), TN (Tarn-Taran), KA (Kapurthala), HR (Hoshiarpur), JR (Jalandhar), SBS (SBS Nagar), RR (Ropar), SAS (SAS Nagar), FH (Fatehgarh Sahib), PA (Patiala), SR (Sangrur), LA (Ludhiana), MA (Moga), BNA (Barnala), MS (Mansa), BA (Bathinda), FT (Faridkot), FR (Ferozpur), MR (Muktsar), and FA (Fazilka).

The Punjab state is approximately bounded by latitude 29°30′N and latitude 32°30′N and longitude 73°50′E and longitude 77°00′E (Fig. 1), covering a total area of 50 362 km^2^ (1.57% of the total land area of India). The study area shares its boundaries with Jammu and Kashmir, Himachal Pradesh, Rajasthan and Pakistan in North, East, South and West (Fig. 1). Major areas of the state are covered with alluvial plains. The unpredictable rainfall is mainly due to monsoon and western disturbances. The data of area, production and yield of rice are acquired from the Special Data Dissemination Standard Division, Directorate of Economics & Statistics, Ministry of Agriculture and Farmers Welfare, Government of India. The data are obtained district-wise for the period 2000–2020 and used for the estimation of atmospheric emissions.

An exhaustive account for greenhouse gases and other air pollutants such as particulate matter (PM_2.5_ and PM_10_), carbon dioxide (CO_2_), carbon monoxide (CO), sulphur dioxide (SO_2_), nitrogen oxides (NO_*x*_), ammonia (NH_3_), methane (CH_4_), volatile organic compounds (VOCs), elemental carbon (EC), organic carbon (OC), black carbon (BC), and polycyclic aromatic hydrocarbons (PAH) is prepared for 2 decades using the following equation:

1 Emissions = *P* × *B* × *C* × *D* × *E* × *F*

where *P* = production of rice, *B* = production-to-crop residue ratio, *C* = dry matter fraction, *D* = fraction burnt, *E* = fraction oxidised, and *F* = emission factors.

Various parameters are given in SI Table S1 based on the earlier studies.^26–29^ The burnt fraction as per IPCC is 25%; however, for this study, the fraction burnt is considered as 80%. Furthermore, we have considered six 1° × 1° grids in the study area (Fig. 1) to compare the satellite data and estimated emissions. The data used for comparison are downloaded through the NASA Giovanni portal (https://giovanni.gsfc.nasa.gov/giovanni/).

The scenario-based future projections are estimated using the following assumptions:

* The mono-cropping system continues at the current pace.

* The area under cultivation is converted to area under rice during rabi season. In other words, crops such as cotton and maize are no longer under cultivation as the rice crop has higher revenue returns.

## Rice crop productions during 2000–2020

**Fig. 2:**
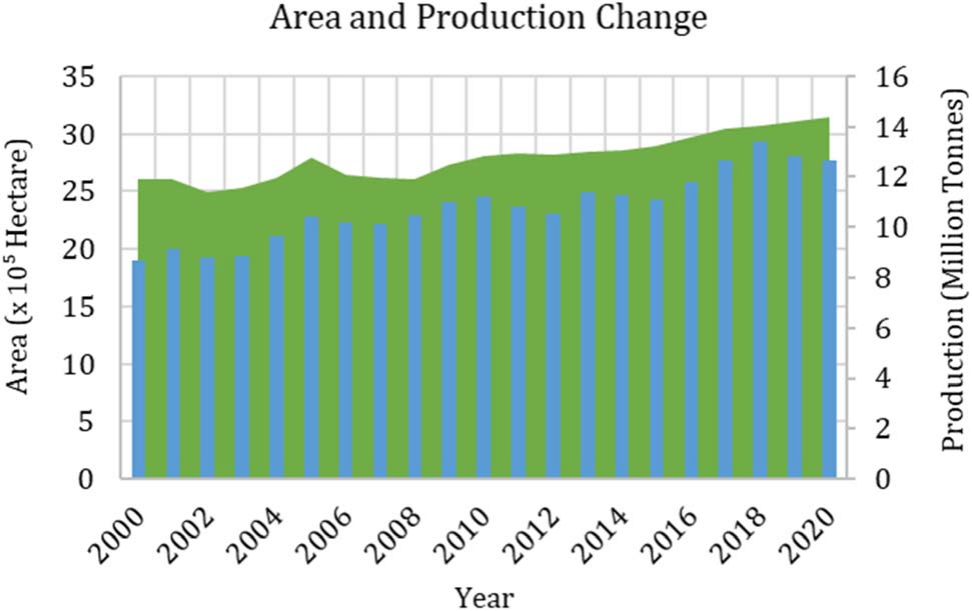
Area and production trend of rice crop in Punjab (India).

Recently, farmers have started sowing high-yielding varieties of paddy crops in the Punjab state, mainly due to the incentives such as free electricity and MSPs (minimum support price) given to the farmers to increase the production of paddy crop. The high water demand of the paddy crop has led to the over-exploitation of groundwater, and the less harvesting and sowing window between rice and wheat crops resulted in burning the rice residue in the field. Our results show that area under paddy crop increased from 260 400 hectares in 2000 to 3 142 000 hectares in 2020. This increased production by 45.42% in the two decades (Fig. 2). Moreover, the yield increased from 56.64 tonnes per hectare in 2000 to 79.55 tonnes per hectares in 2010 and 86.49 tonnes per hectares in 2020.

## District-wise production of rice

Our study shows that although rice production has made Punjab the 3rd largest contributor to the country's rice pool, at the district level, most districts show an increasing trend of area under rice and therefore production. The magnitude of increase is especially noticed in central and south-western districts. For instance, the rice yield increased by 47.19% during 2000–2020 in Sangrur. In the Rupnagar district, the crop yield increased by 60% during 2000–2020. The production in the Bathinda district increased by 94.90% during 2000–2020. The production of rice crop increased 11.34% during 2000–2010, 50.93% during 2010–2020 and 68.04% during 2000–2020 in Mansa. The rice production increased by 110.18% during 2000–2020 in Moga. The rice crop production increased by 134.20% during 2000–2020 in Muktsar. However, few districts showed complex trends such as stagnation in one decade and rise or fall in another. However, the gross trend in Punjab is on the rise. A summary of the decadal change in each district is given in SI Table S2.

Crop residue burning is a major contributor to air pollution, releasing harmful pollutants that degrade air quality and pose health risks. Key emissions include particulate matter (PM_2.5_ and PM_10_), which can penetrate deep into the lungs, and carbon monoxide (CO), which reduces oxygen supply in the blood. Additionally, volatile organic compounds (VOCs) contribute to ozone formation, while polycyclic aromatic hydrocarbons (PAHs) are known carcinogens. Crop burning also releases greenhouse gases like CO_2_, CH_4_, and N_2_O, contributing to climate change. Besides crop residue burning, these pollutants originate from industrial emissions, vehicular exhaust, domestic cooking with solid fuels, and open waste burning. Industries and power plants emit large amounts of PM, CO, and VOCs, while vehicle exhaust, especially from diesel engines, is a significant PM_2.5_ source. Household activities like cooking with biomass also contribute to indoor air pollution. Construction activities and road dust further worsen air quality by increasing the PM levels. The combined effect of these sources leads to severe environmental and health impacts, particularly in regions with intensive agriculture. Reducing these emissions requires promoting sustainable farming practices, improving waste management, and enforcing stricter pollution control measures. The following sections report individual pollutants in the three regions of the study area.

Crop residue burning releases large amounts of particulate matter, BC, and OC. These pollutants influence regional air quality and atmospheric chemistry, drive climate change and affect human health. For instance, long-term exposure can cause asthma, emphysema, bronchitis, irritation of eyes, opacity of corneas and skin irritation.^30–32^ Our study shows that in Doaba, BC emissions increased from 747.41 Mt in 2000 to 1181.46 Mt in 2020. In the Majha region, they increased from 1034.78 Mt in 2000 to 1352.15 Mt in 2020. In the Malwa region, it is observed that BC emissions increased from 4029.11 Mt in 2000 to 5917.31 Mt in 2020. In the Doaba region, OC emissions were estimated as 3574.59 Mt in 2000, 4999.96 Mt in 2010, and 5650.47 Mt in 2020. In the Majha region, OC emissions increased from 4948.94 Mt in 2000 to 5328.41 Mt in 2010 and 6466.79 Mt in 2020. In the Malwa region, OC emissions increased from 19 269.64 Mt in 2000 to 25 500.46 Mt in 2010 to 28 300.19 Mt in 2020.

**Fig. 3:**
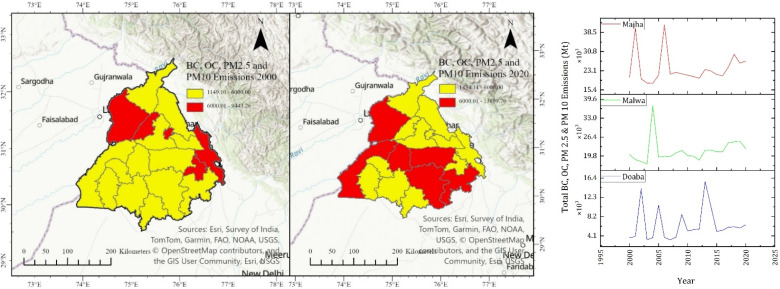
District-wise emission estimation of BC, OC, PM_2.5_ and PM_10_.

Particulate matters emitted from crop residue burning contribute significantly to climate change by sunlight absorption and affecting temperature. Our study shows that PM_2.5_ emissions have increased from 8990.64 Mt in 2000 to 14 211.79 Mt in 2020 in the Doaba region. In the Majha region, PM_2.5_ emissions increased from 12 447.35 Mt in 2000 to 16 264.96 Mt in 2020. In the Malwa region, PM_2.5_ emissions increased from 48 466.05 Mt in 2000 to 71 179.25 Mt in 2020. In the Doaba region, PM_10_ emissions increased from 9857.21 Mt in 2000 to 15 581.60 Mt in 2020. In the Majha region, PM_10_ emissions increased from 13 647.1 Mt in 2000 to 17 832.67 Mt in 2020. In the Malwa region, PM_10_ emissions increased from 53 137.48 Mt in 2000 to 78 039.90 Mt in 2020. The district-wise emissions estimated are described in Fig. 3 for years 2000 and 2020.

The temporal variation shows that emissions have increased dramatically in the state. There is an increased amount of pollution in the Malwa region, which consistently emerges as the dominant emission hotspot across all pollutant classes, while Doaba and Majha exhibit lower but rising emission burdens. Carbonaceous aerosols and particulate matter show a marked escalation over time, with a clear expansion of high-emission districts by 2020. There is an increased amount of black carbon, organic carbon, PM_2.5_, and PM_10_ in the Malwa region, as shown in Fig. 3, reflecting its intensive rice–wheat cropping system and widespread residue burning practices. The accompanying time-series further indicate sustained growth punctuated by episodic peaks, highlighting the years of intensified burning activity. In contrast, Doaba shows lower absolute emissions but greater inter-annual variability, suggesting sensitivity to localized agricultural practices. The intensification of the rice–wheat cropping system and unsustainable agricultural practices are clearly observed as a precursor to emissions observed and estimated in the study. This shows intense burning episodes in the Malwa region as compared to Doaba and Majha.

## Greenhouse gas emissions

In addition to particulate matter and carbonaceous aerosols, large quantities of greenhouse gases are also emitted during crop residue burning. Literature suggests that each year 16% of rice crop residue is converted to ash, which then generate greenhouse gases such as CO_2_, CH_4_, and NO_*x*_.^26^ The agriculture sector shows 17–32% of GHG emissions in India, and the Indian agriculture sector contributes nearly 12% of GHG emissions of the world.^33^ The GHG emissions have a tectonic effect on climate since they are long lived and burning from every year accumulates in the atmosphere. For instance, CO_2_ has a lifetime that can range from 50 to 200 years. Similarly, N_2_O has approximately a lifetime of 120 years. However, CH_4_ has a shorter lifetime than the two but is 28 times more effective in climate warming than CO_2_. Hence, the persistent burning can have alarming effect on the climate, and therefore, it is crucial to control open biomass burning.

**Fig. 4:**
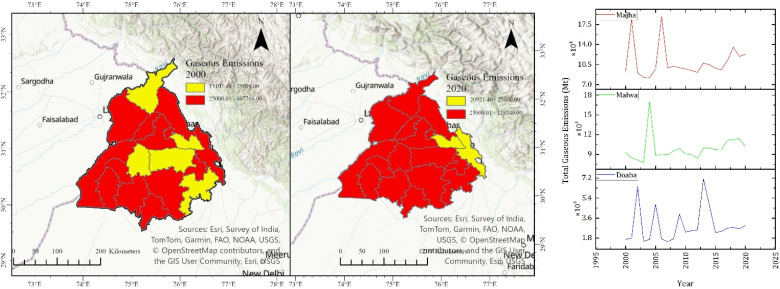
District-wise emission estimation of CO_2_, CH_4_, and N_2_O.

The results of our study show that in the Doaba region, CO_2_ emissions have increased from 1 641 062 Mt in 2000 to 2 594 079.73 Mt in 2020. CH_4_ emissions increased from 2924.67 Mt in 2000 to 4623.11 Mt in 2020. N_2_O increased from 519.94 Mt in 2000 to 821.89 Mt in 2020 in the Doaba region. Similarly, in the Majha region, the emission of CO_2_ is estimated to increase from 2 272 015.65 Mt in 2000 to 2 968 845.19 Mt in 2020. CH_4_ emissions increased from 4049.14 Mt in 2000 to 5261.01 Mt in 2020. N_2_O emissions increased from 719.85 Mt in 2000 to 940.62 Mt in 2020 in the Majha region. The highest concentrations are observed in the Malwa region. It is estimated that CO_2_ emissions increased from 8 846 514.55 Mt in 2000 to 12 992 357.5 Mt in 2020. CH_4_ emissions increased from 15 766.07 Mt in 2000 to 23 154.70 in 2020. N_2_O emissions increased from 2802.86 Mt in 2000 to 4116.39 Mt in 2020 in Malwa. Literature reports that residue burning results in 171 374 Gg per year of CO_2_ emissions, 706.76 Gg per year of CH_4_ emissions and 73.35 Gg per year of N_2_O emissions.^34^ The district-wise emissions estimated are described in Fig. 4 for the years 2000 and 2020.

The decadal temporal variations of greenhouse gases are similar to that of particulate matter and carbonaceous aerosols. A similar spatial pattern is observed for greenhouse gas emissions, where the Malwa region shows an increased amount of CO_2_, CH_4_, and N_2_O emissions, as shown in Fig. 4. The temporal trends indicate a persistent upward trajectory over the two decades, demonstrating that residue burning acts as a chronic source of agricultural greenhouse gases rather than a short-lived seasonal phenomenon.

## Gaseous emissions (sulphur dioxide, carbon mono-oxide, nitrogen oxide and ammonia)

Gaseous emissions can strongly influence regional climate by influencing the atmospheric chemistry. They act as a precursor to secondary aerosol and ozone formation. The SO_2_ concentration can be mainly attributed to fossil fuel burning; however, its concentration peak coincides with crop residue burning season.^21,22^ Our study shows that SO_2_ emissions increased from 433.28 Mt in 2000 to 684.90 Mt in 2020 in the Doaba region. Its emissions increased from 599.87 Mt in 2000 to 783.85 Mt in 2020 in the Majha region. It increased from 2335.71 Mt in 2000 to 3430.33 Mt in 2020 in the Malwa region. Nitrogen compounds such as proteins in the crop residue upon burning releases NH_3_.^35^ It can lead to particulate matter formation, ground-level ozone formation and acid rain upon reacting with SO_2_ and NO_2_. Our study reports that NH_3_ emissions in the study area have increased from 1408.17 Mt in 2000 to 2225.94 Mt in 2020 in the Doaba region. It increased from 1949.58 Mt in 2000 to 2547.52 Mt in 2020 in Majha. Its emissions increased from 7591.07 Mt in 2000 to 11 148.56 Mt in 2020 in the Malwa region. Studies have shown that NH_3_ emissions have increased approximately 30% between 2003 and 2017.^34^

**Fig. 5:**
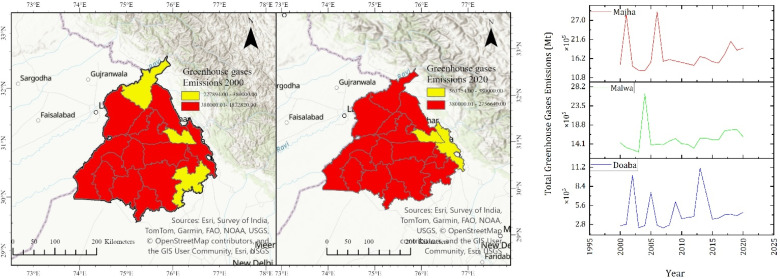
District-wise emission estimation of CO, SO_2_, NO_2_ and NH_3_.

CO is a resultant of incomplete combustion and can have significant effects on air quality and human health. Large-scale crop residue burning results in high concentrations of CO, which can contribute to elevated levels of fine particulate matter and ground-level ozone. CO emissions increased from 100 738.51 Mt in 2000 to 159 240.54 Mt in 2020 in the Doaba region. CO emissions increased from 139 470.27 Mt in 2000 to 182 245.94 Mt in 2020 in Majha. Its emissions increased from 543 053.37 Mt in 2000 to 797 550.67 Mt in 2020 in the Malwa region. NO_*x*_ can have a detrimental effect on the environment by the formation of ozone and fine particulate matter. It also results in the formation of smog and acid rain, and can contribute to climate change. NO_*x*_ emissions increased from 4148.69 Mt in 2000 to 6557.97 Mt in 2020 in the Doaba region. NO_*x*_ emissions increased from 5743.78 Mt in 2000 to 7505.40 Mt in 2020 in Majha. NO_*x*_ emissions increased from 222 364.46 Mt in 2000 to 32 845.37 Mt in 2020 in the Malwa region. It has been reported that 19.8 Gg of NO_*x*_ is emitted per year in Punjab from crop residue burning.^36^ The district-wise emission of gaseous pollutants shows regional disparity (Fig. 5), where the Malwa region shows an increased amount of SO_2_, CO, NO_*x*_, and NH_3_ emissions, with the Majha region exhibiting elevated levels in several districts such as Gurdaspur and Pathankot. However, there are few districts such as Rupnagar in the Malwa region where emissions have decreased. The rising trends reflect enhanced incomplete combustion and nitrogen-rich residue burning, contributing to secondary aerosol formation and photochemical smog. These emissions have important implications for downwind air quality, given the strong influence of prevailing winds during the post-monsoon burning season.

## NMVOC and PAH emissions

PAHs have a long-life span, and therefore, they can remain in the atmosphere for a long period of time, which can result in adverse effects on the environment and human health. In addition to vehicular and industrial emissions, crop residue is a major source of emissions in the atmosphere. In this study, it is observed that, PAHs have increased from 5.42 Mt in 2000 to 8.56 Mt in 2020 in the Doaba region. Their emissions have increased from 7.50 Mt in 2000 to 9.79 Mt in 2020 in the Majha region. The highest emissions have been observed in the Malwa region, and they have increased from 29.20 Mt in 2000 to 42.88 Mt in 2020.

NMVOCs also lead to the formation of ground-level ozone and secondary organic aerosols, which can cause respiratory diseases and result in the formation of smog causing severe damage to air quality. It is estimated in our study that NMVOC emissions increased from 17 006.4 Mt in 2000 to 26 882.54 Mt in 2020 in the Doaba region. NMVOC emissions increased from 23 544.98 Mt in 2000 to 30 766.25 Mt in 2020 in Majha. NMVOC emissions increased from 91 676.75 Mt in 2000 to 134 640.27 Mt in 2020 in Malwa. Other studies have also reported the release of these carcinogenic compounds from crop residue burning.^37^

**Fig. 6:**
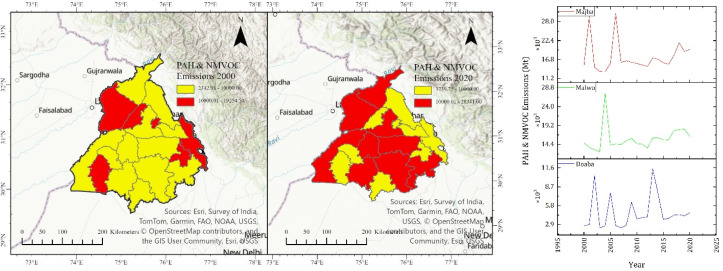
District-wise emission estimation of PAHs and NMVOCs.

Toxic and reactive organic pollutants exhibit comparable trends. There is an increased amount of PAH and NMVOC emissions in the Malwa region, as shown in Fig. 6, with a clear surge for high-emission districts by 2020. The temporal evolution indicates a steady accumulation of these compounds, which are known precursors of secondary organic aerosols and ground-level ozone and pose significant long-term health risks due to their persistence and carcinogenicity. These pollutants are precursors to secondary aerosols and ground-level ozone. Furthermore, they implicate human health as they are carcinogenic and long-lived. They are also responsible for smog formation. This demonstrate that crop residue burning has evolved into a persistent, spatially concentrated, and multi-pollutant source of atmospheric pollution, with the Malwa region acting as the principal driver of emissions across Punjab.

Altogether, it is established that the Malwa region has the highest emissions for all pollutants: GHG, gaseous, particulate matter and toxic reactive organic pollutants. Furthermore, the dispersion of all pollutants largely depends on wind speed, direction and pressure. As a result, the air quality of neighbouring states such as Haryana. Delhi, Rajasthan, and Uttar Pradesh is widely affected by crop residue burning, during the Kharif season. The emissions are dominant during rice harvest as the residue management activities are rarely practiced for rice crop residue. Our study shows that persistent cumulative and spatially concentrated emissions from the state can adversely affect regional climate. The consistent co-occurrence of elevated particulate matter, gaseous pollutants, toxic organics, and greenhouse gases emphasizes the need for region-specific mitigation strategies, rather than uniform state-wide interventions, to effectively address both air quality degradation and climate impacts.

## Correlation variations

The National Green Tribunal has imposed a ban on burning the crop residue due to the adverse environmental impacts. However, lack of awareness, labour shortage, lack of processing facilities, and short window for sowing a rabi season crop, *i.e.*, wheat, force farmers to burn the field.^2^ Henceforth, it is reported that fire counts are observed over Punjab after rabi season (November to May) and kharif season (June to October) harvest every year.^38^ The high intensity and spatial density across the study area cause smog and haze in neighbouring states due to the long-range transport of pollutants and low temperatures during kharif season harvest.

We have considered the active fire count data for the year 2023 from the MODIS FIRMS standard active fire product. The satellite data show that approximately 34 000 active fires were captured during the 2023 kharif season. The highest number of fires, *i.e.*, 27 275, was reported in the Malwa region, followed by 3580 fires in the Majha region and the lowest number was reported in the Doaba region *i.e.*, 2605. Furthermore, district-wise analysis shows that the highest number of fires was reported in the Sangrur (5520) district and lowest in Pathankot (15). Similar trends have been reported in earlier studies, and the highest and lowest fire counts are in Sangrur and Pathankot, respectively, during 2017–18.^3^ Moreover, it is reported that central and southern districts observe the highest number of fires during kharif harvest due to high residue generation.^39^

**Fig. 7:**
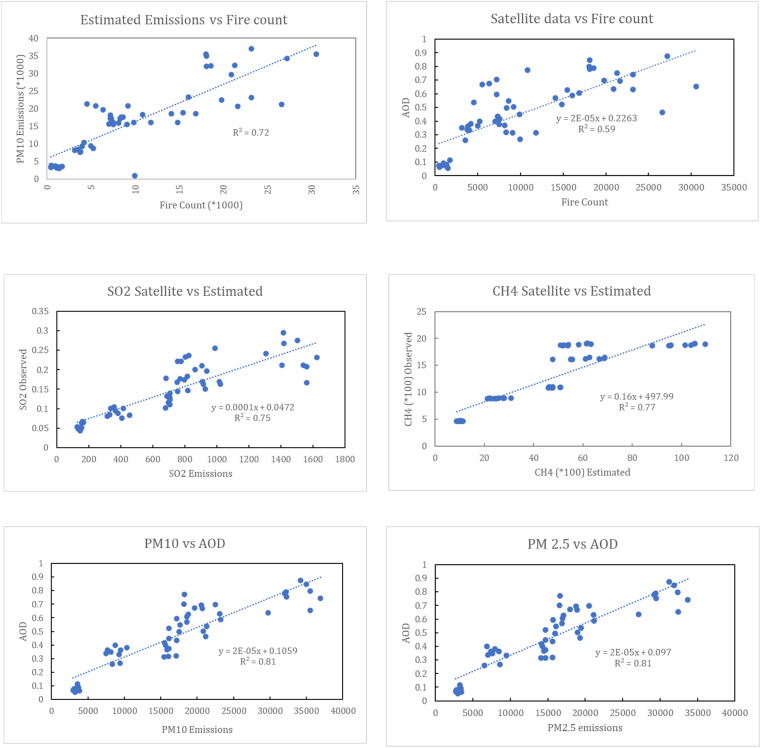
(a) Correlation of estimated emissions and fire counts (2012–2020). (b) Correlation of satellite-observed pollution *vs.* fire counts 2012–2020. (c) Satellite *vs.* estimated emissions of SO_2_. (d) Satellite *vs.* estimated emissions of CH_4_. (e) Correlation between PM_10_ emissions and satellite-observed AOD. (f) Correlation between PM_10_ emissions and satellite-observed AOD.

In addition, we obtained archive fire count data from the MODIS FIRMS archive fire product for the study area, which is available since 2012. These data from 2012 to 2020 are correlated with the emissions estimated (using emission factors as mentioned in previous sections) from the production of rice crop in the study area for the same period. The statistical relationships between fire activity, estimated emissions, and satellite-derived atmospheric indicators demonstrate a strong and coherent linkage between crop residue burning and regional air-quality degradation (Fig. 7). A clear positive association is observed between fire counts and the estimated particulate emissions, confirming the robustness of the emission inventory against independent fire activity data. There is a strong increase in PM_10_ emissions with increasing fire counts, as shown in Fig. 7a, with a high coefficient of determination (*R*^2^ = 0.72). This relationship indicates that variations in particulate emissions are largely explained by the intensity of burning activity, highlighting fire count as a reliable proxy for emission magnitude. The positive slope further suggests a near-linear scaling between fire occurrences and PM_10_ release during crop residue burning events. A similar relationship is evident between satellite-derived aerosol optical depth (AOD) and fire count, as shown in Fig. 7b, where increasing fire activity corresponds to the elevated atmospheric aerosol loading (*R*^2^ = 0.59). Although the correlation is weaker than that for PM emissions, likely due to meteorological influences such as wind dispersion and boundary-layer dynamics, the trend clearly demonstrates that enhanced burning activity translates into higher columnar aerosol concentrations detectable from space. The strong correlation between PM_2.5_ and AOD and PM_10_ and AOD suggests that particulate emissions from crop residue burning are transferred to a higher atmospheric level. The stronger correlation among the two being that of PM_2.5_ shows a longer residence time of finer particles than that of coarse particles.

Further, the estimated values for SO_2_, CH_4_, PM_2.5_, and PM_10_ emissions were correlated with satellite observations. The relationship between satellite-observed SO_2_ concentrations and estimated SO_2_ emissions demonstrates a strong and statistically robust agreement between bottom-up emission estimates and independent remote-sensing observations (Fig. 7c). The fitted regression exhibits a strong positive slope with a high coefficient of determination (*R*^2^ = 0.75), suggesting that a substantial fraction of the observed variability in atmospheric SO_2_ can be explained by the estimated emissions. This close correspondence validates the attribution of enhanced SO_2_ loading to residue burning activities, particularly during periods of intensified fire activity. Minor scatter around the regression line likely reflects the influence of meteorological dispersion, chemical transformation, and background SO_2_ contributions from non-agricultural sources.

Similar consistency was observed when comparing satellite-observed CH_4_ concentrations with estimated CH_4_ emissions, as shown in Fig. 7d. The strong linear relationship (*R*^2^ = 0.77) indicates that crop residue burning significantly contributes to observed methane enhancements, validating the emission estimates and confirming the role of agricultural fires as a non-negligible source of short-lived climate forcers. The coupling between surface emissions and columnar aerosol properties is further highlighted by the relationships between PM_10_ emissions and AOD (Fig. 7e) and PM_2.5_ emissions and AOD (Fig. 7f). In both cases, strong positive correlations (*R*^2^ = 0.81) are observed, indicating that the increases in fine and coarse particulate emissions are efficiently translated into higher atmospheric aerosol burdens. The slightly stronger coherence for PM_2.5_ reflects the greater atmospheric residence time and optical efficiency of fine particles relative to coarse fractions.

This provides compelling evidence that crop residue burning drives a cascade of linked responses, from increased fire activity to enhanced particulate and gaseous emissions, and ultimately to elevated satellite-observed aerosol and trace gas concentrations. These statistically robust relationships confirm the internal consistency of the emission estimates and demonstrate that residue burning exerts a direct and measurable influence on regional atmospheric composition, reinforcing its role as a dominant driver of post-monsoon air pollution and short-term climate forcing in north-western India.

## Emission quantification using grid-wise satellite data

We considered 6 grid boxes of 1° × 1° dimensions as shown in Fig. 1. We considered various emissions over the boxes through the NASA Giovanni portal. District-wise emission data are converted to grid (box) wise by estimating the ratio of rice crop production in a particular district to the area of the district in a particular box. The satellite data are area averaged, where the gridded data includes neighbouring country and/or state. This applies to Grid 1, Grid 3, Grid 4, and Grid 6 (Fig. 1), which include portions of other states/country. The satellite data were proportionally adjusted based on the area of Punjab within these grids. The results show that emissions from the production and the satellite data demonstrate a strong correlation and can be used for future emission prediction.

**Fig. 8:**
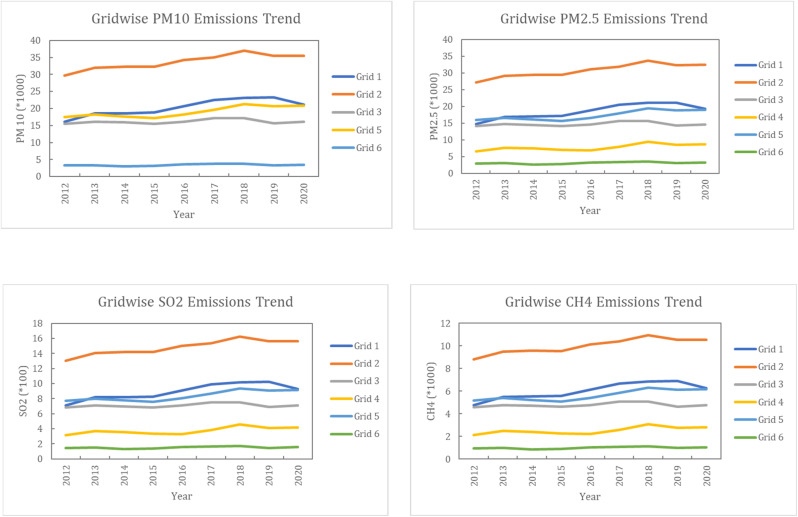
(a) Grid-wise estimated emission trend of PM_10_. (b) Grid-wise estimated emission trend of PM_2.5_. (c) Grid-wise estimated emission trend of SO_2_. (d) Grid-wise estimated emission trend of CH_4_.

**Fig. 9:**
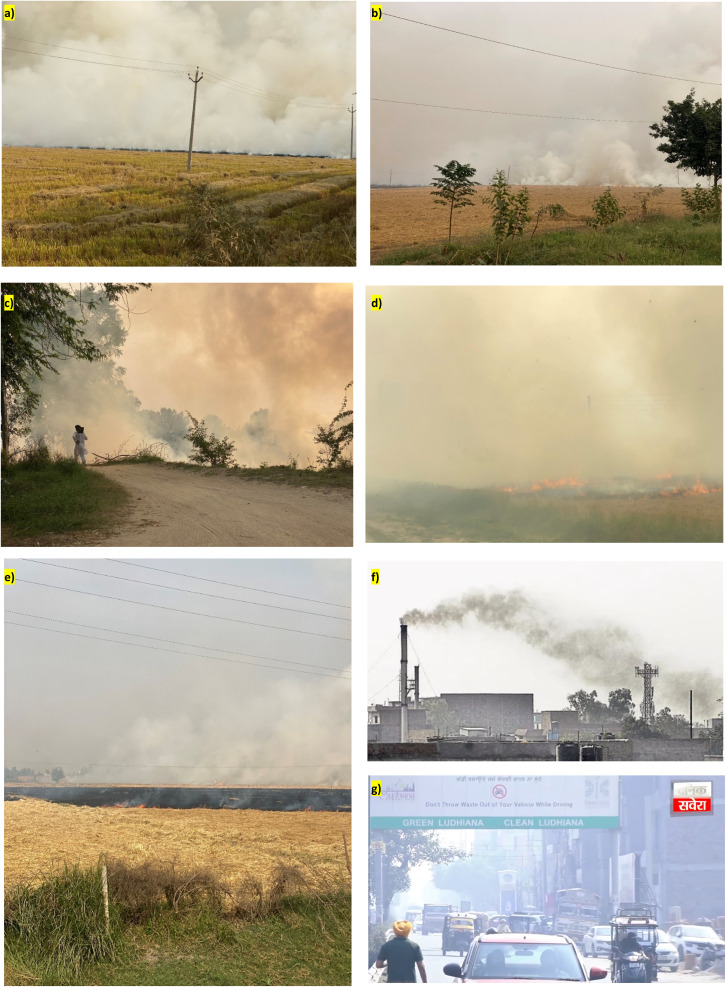
Field photographs showing the extent of pollution in Punjab. (a) Smoke plumes from residue burning are seen near the Bhokra village (around 30.30514°N, 74.96324°E) in the Bathinda district. (b) Active fire blazes and smoke plumes are visible near Bajakhana (around 30.44634°N, 74.9841°E) in the Faridkot district. (c) Smoke from crop residue burning can be seen near the Thuthianwali village (30.04126°N, 75.37853°E) in the Mansa district. (d) Smoke and very poor visibility can be observed near the Dagru village (around 30.83233°N, 75.03036°E) in the Moga district. (e) Scorched land can be seen left after residue burning near the Jalalabad village (around 30.60039°N, 74.21505°E) in the Fazilka district. (f) Smoke plumes from the industrial area (round 30.8827° N, 75.9257° E from Hindustan Times file photo) of the Ludhiana district that has many textile industries and dyeing units. (g) Haze due to pollution was observed on the streets of the Ludhiana city (screengrab from Dainik Savera news aired on 27 Oct 2025).

The grid-wise emission trends show clear spatial heterogeneity and sustained temporal increases in emissions associated with crop residue burning from 2012 to 2020 (Fig. 8a–d). Emission magnitudes consistently differ among grids, indicating the presence of stable emission hotspots rather than uniformly distributed sources. The grid-wise analysis shows that the emissions are non-uniform across the grids with a few grids emerging as hotspots for emissions. The satellite data previously described confirms the presence of hotspot districts in the state. For instance, the highest AOD is observed in grid 2, followed by grid 5 > grid 1 > grid 3 > grid 4, and low AOD is observed in grid 6. This was also observed during field work in these areas, which shows the extent of pollution (Fig. 9). A similar trend can be observed in the estimated concentration of PM_10_ and PM_2.5_ in the study area (Fig. 8a). There is an increase in PM_10_ emissions across all grids, as shown in Fig. 8a, with grid 2 exhibiting the highest levels throughout the study period. Emissions rise steadily until around 2018, followed by slight stabilization toward 2020. Grids 1 and 5 show moderate increases, while grids 3 and 4 remain lower but still exhibit an overall upward trend. A similar pattern is observed for PM_2.5_ emissions, as shown in Fig. 8b, where grid 2 again dominates, followed by grids 1 and 5. The alignment of PM_10_ and PM_2.5_ trends indicates a common combustion source and highlights the growing contribution of fine particulate pollution.

The SO_2_ emission trends, presented in Fig. 8c, show higher concentrations in grid 2, with gradual increases across most grids until 2018 and minor declines thereafter. The spatial correspondence between SO_2_ and particulate emissions suggests that sulphur released during residue burning contributes to secondary aerosol formation. The CH_4_ emission trends, shown in Fig. 8d, further reinforce the spatial persistence of emission hotspots. Grid 2 maintains the highest emissions, while other grids display lower magnitudes but comparable temporal variability, reflecting coherent responses to changes in burning intensity. Overall, these demonstrate that emissions from crop residue burning are spatially clustered and temporally persistent across multiple pollutants. The consistent dominance of specific grids underscores the need for targeted, location-specific mitigation strategies, supported by high-resolution emission inventories.

The distribution of emissions across Punjab shows maximum emission in the south–central area and decreasing magnitudes toward the northern side bordered by Himalayas (Fig. 8a–d). This pattern indicates that emissions are geographically structured and driven by localized agricultural practices. Grid 2, located in the central–southern part of the state and overlapping with the core Malwa region (Fig. 1), consistently records the highest emissions of PM_10_, PM_2.5_, SO_2_, and CH_4_ (Fig. 8a–d). This dominance is linked to intensive rice–wheat cultivation, which produces large quantities of high-silica rice residue that is difficult to manage. The widespread use of mechanized harvesting, combined with limited turnaround time between rice harvest and wheat sowing, promotes open-field burning and results in elevated emissions across multiple pollutants. Grids 1 and 3, situated in southern and south-eastern Punjab, show moderate emission levels with temporal trends similar to grid 2 (Fig. 8a–d). Their spatial proximity and comparable cropping systems suggest shared residue-generation processes, although lower cultivated area and residue loads reduce the overall emission intensity.

In contrast, grid 5, located in the central–northern region corresponding largely to Doaba (Fig. 1), exhibits intermediate emissions, while grids 4 and 6, covering north-western and north-eastern Punjab (Fig. 1), consistently show the lowest emissions (Fig. 8a–d). Although rice–wheat cropping remains prevalent, smaller farm sizes, greater crop diversity, higher proportions of non-agricultural land, and relatively greater adoption of residue management practices resulting in lower residue availability and reduced burning activity likely constrain emission magnitudes compared to the southern grids. Thus, the spatial concentration of emissions in central and southern Punjab underscores the need for targeted mitigation strategies focused on high-residue rice-dominated systems, while differentiated approaches may be sufficient in lower-emission northern grids.

The highest pollution or emissions are observed in grids 1 and 2 and lowest are observed in grid 6. These are directly linked to the production of rice and, consequently, the higher amount of residue. Extensive crop residue burning was observed (Fig. 9a–e) in the districts of Bhatinda, Faridkot, Mansa, Moga and Fazilka that fall in grids 1 and 2, while Ludhiana lies in both grid 2 and grid 5, which shows high pollution due to anthropogenic activities like industries and vast urban areas (Fig. 9f and g). The lower grid-wise pollution can be linked to low increase in rice production and the presence of forest cover in the districts located in the foothills of the Himalayas. For instance, grid 6 shows the lowest emissions and satellite-derived pollutant concentrations. In other words, the maximum concentrations are observed in central and south-western districts. The spatial variation in emissions calls for region-specific mitigation measures that should be focused on high-residue-producing districts.

## Forecast of emissions (CH_4_, SO_2_, AOD, PM_10_ and PM_2.5_)

For future estimation of emissions, the area under rice and the production of rice are estimated using an assumption that the total arable land in 2021–22 as provided by the Ministry of Agriculture and Farmers Welfare, Government of India, is converted into area under rice (*A*) by 2040. This assumption represents upper limit reflecting the consistent increase in rice production due to various socio-economic reasons. Next, to estimate production in the year 2040, the ratio of area under the crop *vs.* production (*P*) of the crop for the past two decades (2000–2020) is used. The area under rice and the *A*/*P* ratio are used to estimate production in the year 2040. This production is used to estimate emissions of various air pollutants using eqn (1). The estimations are calculated in Mt; however, other papers have done the same in Gg or Tg or Mt. Further, three scenarios are considered to estimate emissions based on the fraction of crop residue burnt, *i.e.*, 80% burnt (scenario 1), 50% burnt (scenario 2) and 30% burnt (scenario 3).

### Scenario 1: (80% residue burnt in the field: business as usual)

The results show that if the same scenario continues, *i.e.*, 80% (scenario 1) of residue generated is burnt, the highest increase in emissions would be observed in the Hoshiarpur district (116.71%), followed by the Rupnagar district (95.66%) and Firozpur (67.19%). However, the lowest increase in emission is observed in the Kapurthala district (2.11%). The increase in these districts suggests a strong potential for increase in area under rice crop. In other words, currently, these districts have higher crop diversity. However, the district with low increase in emission may reflect saturation in rice cultivation, where rice production or the area under paddy has reached near-maximum capacity, with most of the cultivable land already allocated to rice cultivation. Another study suggests an increase of 45% in CO_2_ equivalent emissions in India by 2050 for a business-as-usual scenario (Ravindra *et al.*,^34^ 2019).

It is estimated that there is a state-wide increase in emissions if the current practice of 80% crop residue burning continues. Hence, open biomass burning is incompatible with long-term air quality improvements. Elevated emissions will result in short-time climate warming, low visibility, and tropospheric ozone formation.

### Scenario 2: moderate burning (50% residue burning)

In scenario 2, the districts Hoshiarpur (35.44%), Rupnagar (22.29%) and Ferozpur (4.50%) show an increase in emissions. However, the remaining districts may observe a decrease in emissions, with the least decrease in Bathinda and the highest decrease in Amritsar.

The results may suggest that in the districts with the highest increase in production, a 50% reduction in residue burning is insufficient to completely suppress air pollution in areas with rapid agricultural expansion. However, following few mitigation measures and reducing the burning by 50% can substantially lower the emissions or air pollution in many areas. Hence, it is articulate from the mixed response that this scenario will reduce the emissions and, consequently, improve air quality. However, this scenario still allows localized emissions and creates hotspots for poor air quality. By dispersion and other metrological parameters, the pollutants may travel to other parts of the region. For instance, the backward and forward trajectories suggest that the winds carry the particulate matter load from Punjab to Delhi-NCR in Oct–Nov (biomass burning season).^21^

### Scenario 3: pugnacious mitigation measures applied (30% residue burnt)

Lastly, in the case of scenario 3, all districts may observe a decrease in emissions with the least decrease in Rupnagar and the highest decrease in Amritsar. This concludes that aggressive mitigation measures will reduce the emissions, and consequently, improve the air quality even if the agricultural practices are intensified.

The results show that the emissions are reduced in the areas with the highest increase in rice production. The state-wide decrease in emissions suggest that limiting the residue burning through *ex situ* or *in situ* technical interventions may lead to improved air quality without suppressing development.

The detailed district-wise concentrations are provided in Tables 1–3 for each scenario.

**Table 1:** District-wise emissions (×1000 Mt) in scenario 1 (80% residue burnt)

	BC (×10^3^)	OC (×10^3^)	OM (×10^3^)	PM_2.5_ (×10^3^)	PM_10_ (×10^3^)	CO_2_ (×10^3^)	CO (×10^3^)	SO_2_ (×10^3^)	NO_*x*_ (×10^3^)	CH_4_ (×10^3^)	NMVOC (×10^3^)	NH_3_ (×10^3^)	N_2_O (×10^3^)	PAH
Amritsar	0.98	4.67	9.62	11.74	12.87	2142.18	130.09	0.57	5.42	3.82	22.20	1.84	0.68	7.07
Bathinda	0.87	4.15	8.55	10.43	11.44	1903.79	115.61	0.50	4.81	3.39	19.73	1.63	0.60	6.28
Faridkot	0.37	1.79	3.69	4.51	4.94	823.09	49.98	0.22	2.08	1.47	8.53	0.71	0.26	2.72
Fatehgarh Sahib	0.31	1.49	3.08	3.76	4.12	685.68	41.64	0.18	1.73	1.22	7.11	0.59	0.22	2.26
Firozpur	1.33	6.36	13.10	15.99	17.53	2919.26	177.28	0.77	7.38	5.20	30.25	2.50	0.92	9.63
Gurdaspur	0.58	2.80	5.76	7.03	7.71	1283.88	77.96	0.34	3.25	2.29	13.30	1.10	0.41	4.24
Hoshiarpur	0.50	2.38	4.90	5.98	6.55	1091.09	66.26	0.29	2.76	1.94	11.31	0.94	0.35	3.60
Jalandhar	0.66	3.15	6.48	7.91	8.68	1444.69	87.73	0.38	3.65	2.57	14.97	1.24	0.46	4.77
Kapurthala	0.37	1.78	3.67	4.48	4.91	817.29	49.63	0.22	2.07	1.46	8.47	0.70	0.26	2.70
Ludhiana	0.96	4.57	9.42	11.50	12.61	2099.32	127.48	0.55	5.31	3.74	21.76	1.80	0.67	6.93
Mansa	0.54	2.59	5.34	6.52	7.15	1190.28	72.28	0.31	3.01	2.12	12.33	1.02	0.38	3.93
Moga	0.62	2.96	6.10	7.45	8.16	1359.32	82.55	0.36	3.44	2.42	14.09	1.17	0.43	4.49
Muktsar	0.65	3.09	6.38	7.78	8.53	1420.81	86.28	0.38	3.59	2.53	14.72	1.22	0.45	4.69
SBS Nagar	0.27	1.27	2.62	3.20	3.51	584.47	35.49	0.15	1.48	1.04	6.06	0.50	0.19	1.93
Patiala	0.81	3.88	7.99	9.75	10.69	1779.64	108.07	0.47	4.50	3.17	18.44	1.53	0.56	5.87
Rupnagar	0.36	1.71	3.53	4.31	4.72	786.19	47.74	0.21	1.99	1.40	8.15	0.67	0.25	2.59
Sangrur	1.47	7.01	14.45	17.63	19.33	3218.66	195.46	0.85	8.14	5.74	33.36	2.76	1.02	10.62

**Table 2:** District-wise emissions (×1000 Mt) in scenario 1 (50% residue burnt)

	BC (×10^3^)	OC (×10^3^)	OM (×10^3^)	PM_2.5_ (×10^3^)	PM_10_ (×10^3^)	CO_2_ (×10^3^)	CO (×10^3^)	SO_2_ (×10^3^)	NO_*x*_ (×10^3^)	CH_4_ (×10^3^)	NMVOC (×10^3^)	NH_3_ (×10^3^)	N_2_O (×10^3^)	PAH
Amritsar	0.61	2.92	6.01	7.34	8.04	1338.86	81.30	0.35	3.38	2.39	13.87	1.15	0.42	4.42
Bathinda	0.54	2.59	5.34	6.52	7.15	1189.87	72.26	0.31	3.01	2.12	12.33	1.02	0.38	3.93
Faridkot	0.23	1.12	2.31	2.82	3.09	514.43	31.24	0.14	1.30	0.92	5.33	0.44	0.16	1.70
Fatehgarh Sahib	0.20	0.93	1.92	2.35	2.57	428.55	26.02	0.11	1.08	0.76	4.44	0.37	0.14	1.41
Firozpur	0.83	3.97	8.19	10.00	10.96	1824.54	110.80	0.48	4.61	3.25	18.91	1.57	0.58	6.02
Gurdaspur	0.37	1.75	3.60	4.40	4.82	802.42	48.73	0.21	2.03	1.43	8.32	0.69	0.25	2.65
Hoshiarpur	0.31	1.49	3.06	3.74	4.10	681.93	41.41	0.18	1.72	1.22	7.07	0.59	0.22	2.25
Jalandhar	0.41	1.97	4.05	4.95	5.42	902.93	54.83	0.24	2.28	1.61	9.36	0.77	0.29	2.98
Kapurthala	0.23	1.11	2.29	2.80	3.07	510.80	31.02	0.13	1.29	0.91	5.29	0.44	0.16	1.69
Ludhiana	0.60	2.86	5.89	7.19	7.88	1312.07	79.68	0.35	3.32	2.34	13.60	1.13	0.42	4.33
Mansa	0.34	1.62	3.34	4.08	4.47	743.93	45.18	0.20	1.88	1.33	7.71	0.64	0.24	2.46
Moga	0.39	1.85	3.81	4.65	5.10	849.58	51.59	0.22	2.15	1.51	8.80	0.73	0.27	2.80
Muktsar	0.40	1.93	3.99	4.86	5.33	888.00	53.92	0.23	2.24	1.58	9.20	0.76	0.28	2.93
SBS Nagar	0.17	0.80	1.64	2.00	2.19	365.29	22.18	0.10	0.92	0.65	3.79	0.31	0.12	1.21
Patiala	0.51	2.42	4.99	6.09	6.68	1112.28	67.54	0.29	2.81	1.98	11.53	0.95	0.35	3.67
Rupnagar	0.22	1.07	2.21	2.69	2.95	491.37	29.84	0.13	1.24	0.88	5.09	0.42	0.16	1.62
Sangrur	0.71	3.38	6.96	8.49	9.31	1550.42	94.15	0.41	3.92	2.76	16.07	1.33	0.49	6.64

**Table 3:** District-wise emissions (Mt) in case of scenario 3 (30% residue burnt)

	BC (×10^3^)	OC (×10^3^)	OM (×10^3^)	PM_2.5_ (×10^3^)	PM_10_ (×10^3^)	CO_2_ (×10^3^)	CO (×10^3^)	SO_2_ (×10^3^)	NO_*x*_ (×10^3^)	CH_4_ (×10^3^)	NMVOC (×10^3^)	NH_3_ (×10^3^)	N_2_O (×10^3^)	PAH
Amritsar	0.37	1.75	3.61	4.40	4.83	803.32	48.78	0.21	2.03	1.43	8.32	0.69	0.25	2.65
Bathinda	0.33	1.56	3.20	3.91	4.29	713.92	43.35	0.19	1.80	1.27	7.40	0.61	0.23	2.36
Faridkot	0.14	0.67	1.39	1.69	1.85	308.66	18.74	0.08	0.78	0.55	3.20	0.26	0.10	1.02
Fatehgarh Sahib	0.12	0.56	1.15	1.41	1.54	257.13	15.61	0.07	0.65	0.46	2.66	0.22	0.08	0.85
Firozpur	0.50	2.38	4.91	6.00	6.58	1094.72	66.48	0.29	2.77	1.95	11.34	0.94	0.35	3.61
Gurdaspur	0.22	1.05	2.16	2.64	2.89	481.45	29.24	0.13	1.22	0.86	4.99	0.41	0.15	1.59
Hoshiarpur	0.19	0.89	1.84	2.24	2.46	409.16	24.85	0.11	1.03	0.73	4.24	0.35	0.13	1.35
Jalandhar	0.25	1.18	2.43	2.97	3.25	541.76	32.90	0.14	1.37	0.97	5.61	0.46	0.17	1.79
Kapurthala	0.14	0.67	1.38	1.68	1.84	306.48	18.61	0.08	0.77	0.55	3.18	0.26	0.10	1.01
Ludhiana	0.36	1.71	3.53	4.31	4.73	787.24	47.81	0.21	1.99	1.40	8.16	0.68	0.25	2.60
Mansa	0.20	0.97	2.00	2.45	2.68	446.36	27.11	0.12	1.13	0.80	4.63	0.38	0.14	1.47
Moga	0.23	1.11	2.29	2.79	3.06	509.75	30.95	0.13	1.29	0.91	5.28	0.44	0.16	1.68
Muktsar	0.24	1.16	2.39	2.92	3.20	532.80	32.35	0.14	1.35	0.95	5.52	0.46	0.17	1.76
SBS Nagar	0.10	0.48	0.98	1.20	1.32	219.17	13.31	0.06	0.55	0.39	2.27	0.19	0.07	0.72
Patiala	0.30	1.45	3.00	3.66	4.01	667.37	40.53	0.18	1.69	1.19	6.92	0.57	0.21	2.20
Rupnagar	0.13	0.64	1.32	1.62	1.77	294.82	17.90	0.08	0.75	0.53	3.06	0.25	0.09	0.97
Sangrur	0.55	2.63	5.42	6.61	7.25	1207.00	73.30	0.32	3.05	2.15	12.51	1.04	0.38	3.98

From the perspective of human health, reductions in crop residue burning through residue management can result in decreased exposure to fine particles. Subsequently, the risk for respiratory diseases and early mortality will reduce significantly in people living across Punjab and its neighbouring states. Similar results were found by another study which concluded that a 1% decrease in fires will substantially improve the air quality.^5^ Furthermore, immediate benefits might be observed for the regional climate, and secondary aerosol formation may also reduce due to the reduction in its precursors.^34^

This scenario-based analysis projects an estimation of emissions that can shape the future air quality in the region and its surroundings. The analysis provides an insight into the current practices and projects the improvements and/or degradation in the near future. The business-as-usual scenario shows that it is imminent to prioritize the mitigation measures. However, scenarios 2 and 3 represent that aggressive mitigation measures are the only way to substantially improve air quality and curb regional and global climate changes.

## Recommendations or alternative methods of residue management

The mitigation of the observed multi-pollutant emission burden from crop residue burning requires an integrated residue-management framework that combines *in situ*, *ex situ*, and policy-supported interventions. *In situ* approaches such as mechanical incorporation, zero-tillage seeding, mulching, and application of microbial bio-decomposers can help retain soil moisture by 5–10%, balance soil temperature during summer and winter seasons^40^ balance soil pH, improve water infiltration rate of soil and provide essential nutrients to the soil.^41^ This substantially reduces open combustion while enhancing soil organic carbon content, moisture retention, and nutrient recycling, thereby indirectly lowering NH_3_ and N_2_O volatilization.^42–44^ Complementary *ex situ* utilization pathways—including biomass pelletization, bioenergy generation, controlled pyrolysis for biochar production, and industrial reuse in paper or composite materials—offer economically viable alternatives that convert residues into value-added products while reducing emissions of PM_2.5_, BC, NMVOCs, and greenhouse gases.^34,45,46^ Rice crop residue has multi-fold benefits to be used in the power sector since it has the merits of high calorific value, low ash content, ready availability, and nearly 100% efficiency when boiler burnt. This can provide additional income to farmers, and is also environmentally friendly as compared to coal-fed electricity generation.^47–50^

These technological options should be reinforced through incentive-based policies, decentralized biomass aggregation systems, and improved satellite-based monitoring to ensure large-scale adoption and accurate emission accounting.^34,42,45,51^ In the longer term, agro-ecological measures such as crop diversification, adoption of short-duration cultivation, and integrated nutrient-residue cycling can structurally reduce residue loads and associated nitrogen-rich emissions. Collectively, a hybrid strategy that links farm-level management with circular bioeconomy utilization and governance support provides the most effective pathway for simultaneous air-quality improvement, climate mitigation, and agricultural sustainability.

## Limitations of the study

There are few limitations of the study, such as the satellite data used in the study to validate the estimated emissions has a coarse spatial resolution. However, the unavailability of ground monitoring stations creates a unique challenge for such studies (Table 4). The production data used for forecast can fluctuate from the real-life scenario because the production can be increased by various enhancing methods such as chemical fertilizers, and the area under other land uses such as forests and wetlands can also be used for crop production. Henceforth, the only method to ensure air quality improvement is to adopt sustainable methods of residue management and proper implementation of policies, rules and acts to curb residue burning.

**Table 4:** Challenges faced in emission research and possible solutions

Challenges	Possible solutions
Accurate and reliable quantification of emissions from crop residue burning	High resolution inventories using field experiments and remote sensing
Distinguishing crop residue burning PM_2.5_ and urban/industrial PM_2.5_	Models for source apportionment during peak burning season
Behavioural resistance of farmers to change	Farmer centric incentives and policies to encourage sustainable agriculture

## Conclusion

It is evident that unsustainable crop residue burning is causing poor air quality in the study area and the surrounding areas. Moreover, the practice of residue burning leads to adverse effects on human health and soil fertility. However, due to various socio-economic conditions, farmers burn the fields. Our study concludes that the highest increase in rice crop production during 2000–2020 can be observed in Muktsar (134.20%) followed by Moga (110.18%) and Bathinda (94.90%). However, the least increase in rice crop production can be observed in Firozpur (10.18%). These districts are located in the Malwa region of the study area. Consequently, the highest increase in emissions can also be seen in these three districts.

Over the two-decade assessment period, all major pollutant classes exhibit statistically significant upward trends, with the Malwa region consistently emerging as the primary emission hotspot. The synchronous rise of SO_2_ and NH_3_ during residue-burning seasons indicates a substantial and previously underappreciated agricultural contribution to regional sulphur loading, secondary particulate formation, and ozone chemistry. Concurrent increases in CO and NO_*x*_ further confirm intensified incomplete combustion processes, strengthening the linkage between crop residue burning, secondary PM_2.5_ generation, and chronic air-quality degradation. The sustained growth of NMVOCs and PAHs highlights escalating toxic and carcinogenic risks that extend beyond episodic pollution events, while pronounced increases in black carbon, organic carbon, and coarse and fine particulates highlight the dual implications for public health and regional climate forcing. Increasing emissions of CO_2_, CH_4_, and N_2_O collectively demonstrate that open-field residue burning is not only a dominant driver of fine particulate pollution but also a non-trivial contributor to the agricultural greenhouse-gas budget, thereby undermining both air-quality management and long-term climate mitigation objectives.

The forecast for the next 2 decades shows that with the same fraction burnt (80%), the highest emissions will be observed in Ferozpur, Rupnagar and Hoshiarpur districts. The least production increase can conclude that the district is already sowing rice crop at its full capacity. This may also indicate that these districts, in order to increase their rice crop production, can utilize area under other land cover such as forests or wetlands, which should be strictly discouraged.

## Conflicts of interest

There are no conflicts to declare.

## Supplementary Material

RA-016-D5RA09439A-s001

## Data Availability

The data will be made available upon reasonable request to the first author. Supplementary information (SI) is available. See DOI: https://doi.org/10.1039/d5ra09439a.
