# Supplementary material for: Long-term variability of air quality and greenhouse gas emissions from rice crop burning in Punjab during 2012–2020
Source: RSC Adv. 2026 Mar 10;16(15):13304–19. doi: 10.1039/d5ra09439a (PMC12973183; doi:10.1039/d5ra09439a)
Supplement: RA-016-D5RA09439A-s001 [file RA-016-D5RA09439A-s001.pdf]

Supplementary Information (SI) for RSC Advances.
This journal is © The Royal Society of Chemistry 2026

For estimation of gases emitting from rice crop residue burning, the following parameters are used as per the literature

Residue to crop Ratio (B) = 1.76; Dry matter fraction (C) = 0.85; Fraction burnt (D) = 0.8; Fraction actually oxidized (E) = 0.89; Emission factors (F)= given in Supplementary table 1 below.

Supplementary table 1: Emission factors for pollutants released during crop residue burning

Pollutant	Emission Factor (g/Kg)
BC	0.69
OC	3.3
OM	6.8
$PM_{2.5}$	8.3
PM_{10}	9.1
CO_2	1515
CO	92
SO_2	0.4
NOx	3.83
CH_4	2.7
NMVOC	15.7
NH_3	1.3
PAH	0.005
N_2O	0.48

Supplementary table 2: district wise area under rice crop for years 2000, 2010 and 2020.

Area Under rice Crop (Hectare)					
			Year		
S.No.	Region	District	2000	2010	2020
1	Majha	AMRITSAR	318468	358697	365452
2		GURDASPUR	198102	203583	192534
3	Malwa	BATHINDA	99017	106673	179091
4		FARIDKOT	89908	101346	115874
5		FATEHGARH SAHIB	82325	81704	86351
6		FIROZEPUR	279552	257760	304810
7		LUDHIANA	236929	257130	259046
8		MANSA	91014	77891	118691
9		MOGA	116710	171675	181307
10		MUKTSAR	91101	111149	189685
11		PATIALA	250770	234373	234791
12		RUPNAGAR	50108	37493	40424
13		SANGRUR	359519	272354	288980
14	Doaba	HOSHIARPUR	61399	72514	78129
15		JALANDHAR	132980	163057	174996
16		KAPURTHALA	99887	117806	120183
17		NAWANSHAHR	45944	57948	60033
